# How Does the Dinoflagellate Parasite *Hematodinium* Outsmart the Immune System of Its Crustacean Hosts?

**DOI:** 10.1371/journal.ppat.1004724

**Published:** 2015-05-07

**Authors:** Andrew F. Rowley, Amanda L. Smith, Charlotte E. Davies

**Affiliations:** Department of Biosciences, College of Science, Swansea University, Swansea, Wales, United Kingdom; The Fox Chase Cancer Center, UNITED STATES

## *Hematodinium* Infections: A Problem for the Sustainability of Crustacean Fisheries and Aquaculture Worldwide

Dinoflagellates of the genus *Hematodinium* infect over 40 species of marine crustaceans. Since the initial description of this parasite in France in the 1930s [1], it appears to have spread globally, causing economic loss in some species, including Tanner crabs (*Chionoecetes bairdi*) in Alaska [2], blue crabs (*Callinectes sapidus*) in the United States [3], edible crabs (*Cancer pagurus*) and Norway lobsters (*Nephrops norvegicus*) in Europe [4–7], sand crabs (*Portunus armatus*) in Australia [8], and Chinese swimming crabs (*Portunus trituberculatus*) in northern China [9]. Because this disease is thought to be fatal, it may be of great significance to the sustainability of both captive shellfish fisheries and aquaculture [10,11]. For instance, the decline in blue crabs from the Atlantic to Gulf Coasts in the US appears to be linked to epizootic outbreaks of disease caused by *Hematodinium* [12]. Similarly, the reduction in velvet swimmer crab (*Necora puber*) numbers in Brittany in the 1980s has been attributed to the high prevalence of such infections [13]. Finally, the recent reports of *Hematodinium* infections in crabs and shrimp raised under aquaculture conditions in China [9,14,15] highlight the danger to additional, cultured crustacean populations.

The number and host range of species of *Hematodinium* is unclear. Chatton and Poisson [1] identified one species of this parasite that they termed *H*. *perezi*. Notably, this species of parasite was reported in two different species of crabs from several locations around the French coast. A second species, *H*. *australis*, was first described in sand crabs from Moreton Bay in Australia [8]. Recent studies making use of the variability of the ITS1 rDNA region of *H*. *perezi* suggest that there are three clades (genotypes), I–III, of this species (see [11] for further details). Pagenkopp Lohan et al. [16] concluded that blue crab populations collected from the Atlantic to the Gulf coasts of the US were all affected by one genotype, *H*. *perezi* (III), which implies a large geographic range. Importantly, other species of crabs collected from the same region were also infected by the same genotype of *H*. *perezi* [16]. In Europe, there may be two clades of *Hematodinium* infecting a range of crabs. *H*. *perezi* genotype I has been reported in *Liocarcinus depurator* [17] and *Carcinus maenas*. The second species of *Hematodinium*, currently unnamed (*Hematodinium* sp.) infects a wider range of crustaceans, including edible crabs (*C*. *pagurus*). Importantly, these observations imply that this parasite is a host generalist and may be able to “jump” from host species to species, thereby making it a significant threat to a wide variety of commercially important decapods.

## What Is the Interaction between the Host and *Hematodinium*?

**Fig 1:**
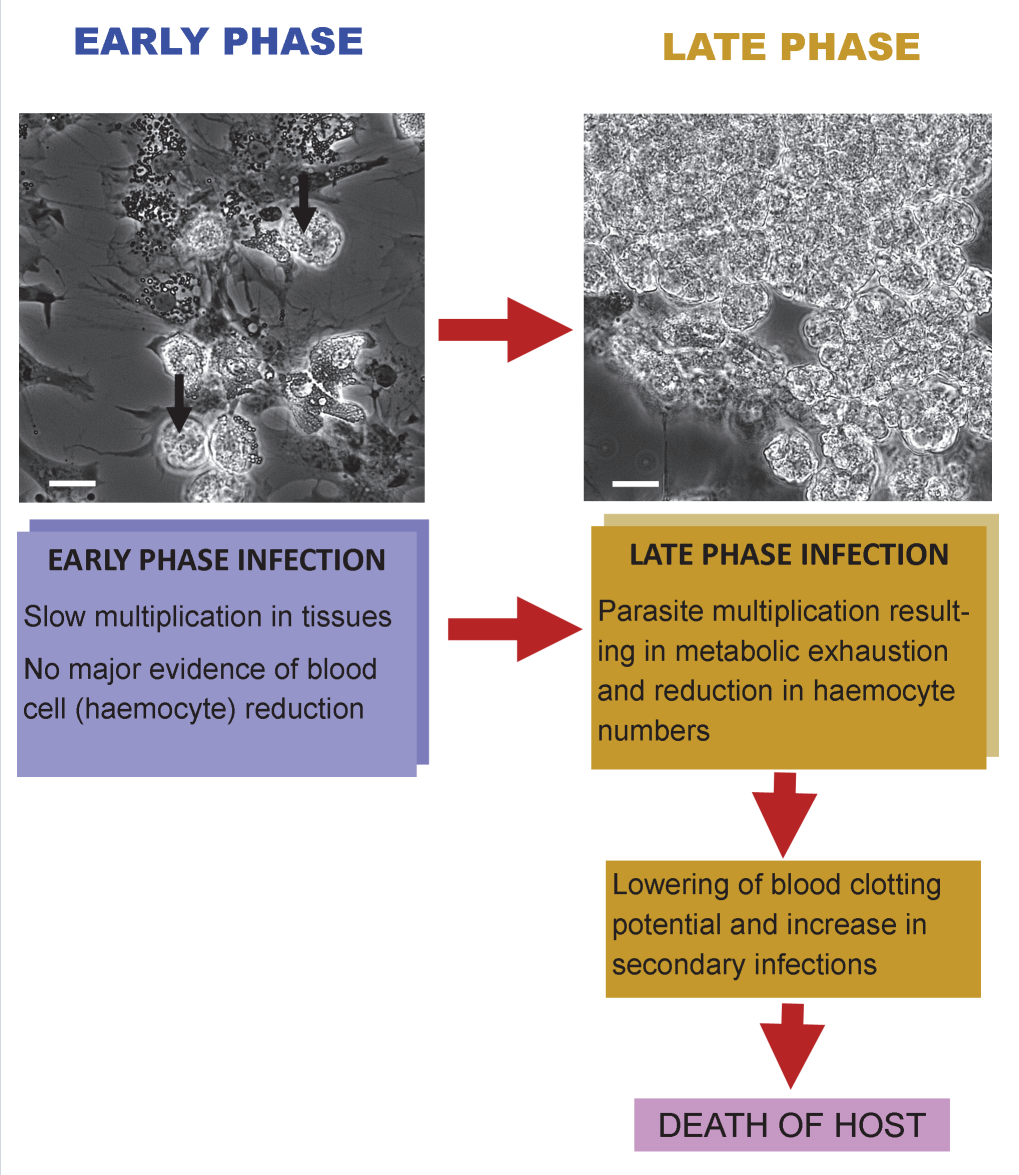
Interaction of *Hematodinium* with its crustacean host. At an early phase of infection there is a small number of parasites in the haemolymph (unlabelled arrows) and a large number of haemocytes in circulation, as found in uninfected individuals (spread cells in micrograph). In late-phase infections, there are often large masses of *Hematodinium* in the haemolymph and very few circulating haemocytes. This reduction in haemocytes results in an increased likelihood of secondary infections and prolonged bleeding times following wounding. Death probably ensues as a result of parasite development and utilisation of host’s metabolic products, as well as a heightened chance of secondary infections.

Reports on the pathology of *Hematodinium* spp. infections in a variety of crustacean hosts are all similar. While the mode of transmission of the parasite to its hosts is unclear, it is thought that susceptible animals may become infected after moulting [18,19], suggesting an integumentary route of invasion when the cuticle is soft and vulnerable. The parasites gain entry to the underlying tissues, including the main body space, termed the haemocoel, and multiply in tissues and organ systems including the heart, hepatopancreas, connective tissue, and the haemolymph (“blood”) [10]. The timescale of infection is protracted in some host species of crustacean, such as those living in colder waters, where the period from infection to death may be several months [2], whereas in others, it can be much more rapid [20]. Early phases of infection (see “Early Phase” in Fig 1) are characterised by limited changes in the number of circulating haemocytes and only modest elevation in parasite abundance in this tissue. One of the hallmarks of advanced *Hematodinium* infections (“Late Phase” in Fig 1) is the marked multiplication of the parasites in all tissues and a simultaneous reduction in the number of circulating haemocytes, leaving the affected animals unable to mount effective cellular defence and haemostatic responses [20]. Accompanying the increase in the number of parasites in advanced infections comes muscle necrosis [21] and “metabolic exhaustion” [22], characterised by significant reduction in plasma proteins, including haemocyanin that serves as both a respiratory pigment and as an immunologically active molecule [23]. Interestingly, these pathological changes may be reflected in alteration in the general behaviour of infected animals [24,25], leaving them more susceptible to predation and loss from the fishery.

## Is There Evidence of Immune Reactivity to the Presence of *Hematodinium* in Crustacean Hosts?

The immune system of invertebrates has been extensively studied, especially in insects and crustaceans. However, our understanding of the interaction between pathogens and parasites and their hosts is less well understood, with the notable exception of those agents that infect humans while in their invertebrate vectors. For a brief description of the main features of the crustacean immune system, the reader is directed to Box 1 in this article and recent reviews [26,27].

**Box 1. The Invertebrate Immune System: A Brief Primer**

The invertebrate immune system consists of cellular and humoral defences that are principally mediated by blood cells, which in arthropods are termed haemocytes. Non-self is recognised by a variety of immune factors, including lectins and products of the prophenoloxidase cascade [26,27]. The main cellular defence reactions are phagocytosis and a process in which pathogens and parasites are walled off, called nodule formation or encapsulation. The blood (haemolymph) contains a wide variety of antimicrobial factors including hydrolytic enzymes (e.g., lysozyme) and antimicrobial peptides (e.g., defensins). Although the defence reactions of invertebrates are considered to be non-specific, recent studies have questioned whether a unique form of immune memory is present at least in some invertebrates [27].

A significant number of studies have examined the pathology of *Hematodinium* infections in a wide range of crustacean hosts (see [10] for review). Although in some cases there has been evidence provided of a cellular host response to this parasite, such as the presence of haemocytic nodules [6], close examination reveals there is little or no evidence for the presence of the various stages of this parasite within the nodules, implying that their generation is not a direct response to the parasite. A potential explanation for the presence of nodules is as a result of other co-infections—which are common in some crustaceans [28–31]—or tissue damage caused by *Hematodinium* in late-phase infections (Fig 1) [31]. One report has produced convincing evidence for the uptake of *Hematodinium* by the fixed phagocytes of *N*. *norvegicus* [32], but to our knowledge no other studies have found such an association.

**Fig 2:**
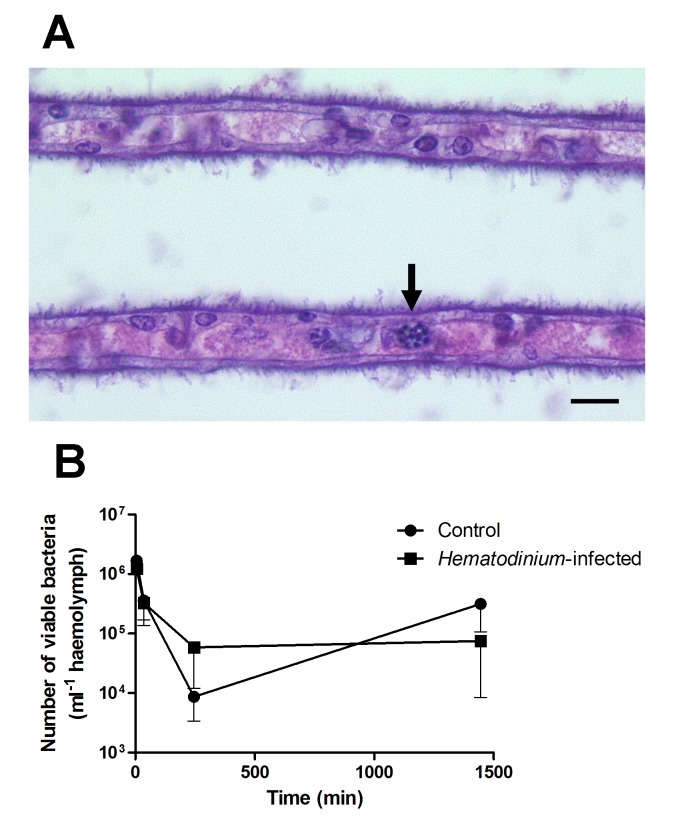
Experimental exploration of the interaction between *Hematodinium* and its host. (A). Histological appearance of gill lamellae of a crab seven days post-injection of *Hematodinium*. Note these parasites in the gill space (unlabelled arrow). Scale bar = 10 μm. (B). Clearance of the gram-positive bacterium, *Bacillus subtilis* from *Hematodinium*-infected (early-stage infection) and uninfected juvenile edible crabs (*C*. *pagurus*). Crabs were injected intrahaemocoelically with 10^5^ live bacteria and the numbers of remaining viable bacteria (cfu) in the circulation determined by standard plate counting on tryptone soya agar + 2% NaCl plates. Values shown are means of cfu with standard error of the mean (SEM), *n* = 5. The values are not statistically different between animals of differing disease status.

To further investigate this apparent lack of host response to the presence of *Hematodinium*, we injected juvenile edible crabs (*C*. *pagurus*) with *Hematodinium* and monitored changes in the haemogramme. These experiments found no evidence for reduction in the number of circulating haemocytes that might have resulted from localised nodule formation, and histological examination of various tissues, including the gills, failed to show the presence of nodules indicative of such a host response. Furthermore, seven days post-challenge, 100% of experimental animals had stages of the *Hematodinium* parasite free in the haemolymph (Fig 2A) implying that they had not been cleared from circulation by either fixed or free phagocytes. These studies support the concept of a failure of the immune defences to recognise and clear these parasites from the haemocoel. Hence, it can be concluded that *Hematodinium* spp., regardless of the host species studied, are not effectively recognised as foreign by the immune system.

## What Are the Potential Mechanisms Employed by *Hematodinium* to Avoid Immune Recognition and Elimination?

There are several recognised strategies employed by parasites and pathogens to successfully colonise their hosts without their elimination by the cellular and humoral components of the immune system (see Box 1). Table 1 summarises and speculates on some of the more likely mechanisms that *Hematodinium* could use, and some of these are now explored.

**Table 1:** An exploration of potential mechanisms employed by *Hematodinium* to avoid, circumvent, or suppress the crustacean immune system.

Mechanism	Evidence	References
Inhibition of haemopoiesis leading to reduction of circulating haemocytes (haemocytopaenia) and lowering of cellular defence and haemostasis	None in early infections in edible crabs (see Fig 1). Later reduction in haemocyte numbers as shown in this figure may result from “metabolic exhaustion” rather than direct suppression of haemopoiesis	[22]
General (non-selective) immune suppression	None and unlikely to be an explanation, from studies of co-infections in which the presence of *Hematodinium* does not appear to influence the ability of other parasites and pathogens to multiply in the host	[26,30,33,34]
Specific evasion or molecular mimicry	There is no evidence of any obvious haemocytic response to the presence of *Hematodinium* in the haemocoel and tissues	e.g. [7,17]
Production of factors that interfere with killing action of plasma and/or haemocytes	Generation of acid phosphatase by *Hematodinium* parasites that may interfere with generation of cytotoxic oxygen radicals in haemocytes. However, changes in oxygen radical generation in parasitized crustaceans has not been explored.	[22,40,41]
Generation of cytotoxic molecules	Some free-living dinoflagellates generate such toxins, but there is no evidence of this in *Hematodinium*	[42,43]

As haemocytes are the cells at the centre of the invertebrate immune system and are also responsible for blood coagulation, any reduction in their numbers has a profound effect on the survival of the infected host. One of the hallmarks of late-phase *Hematodinium* infections is the reduction in the number of circulating haemocytes that leaves the host unable to mount an effective cellular defence and haemostatic response [20]. The paucity of haemocytes in circulation could reflect their loss due to extensive infiltration of infected tissues but there is no evidence for this event from histological studies (e.g. [7]) and it is more likely that it results from suppression of haemopoietic activity. As we have shown that haemocytopaenia is a relatively late response to advanced infections, at least in the edible crab (Fig 1), this implies that the principal mechanism of pathogenicity does not involve suppression of haemopoiesis. Furthermore, we would argue that this loss of haemocytes is probably linked to the state of metabolic exhaustion [20,22] caused by the rapidly multiplying parasites “stealing” host resources, rather than any parasite-driven direct inhibition of haemopoiesis.

Some crustaceans with *Hematodinium* have been found to be susceptible to secondary infections caused by other parasites and pathogens [10,28,29]. One example of this interaction comes from studies with naturally *Hematodinium*-infected crabs that were found to have a secondary fungal infection [28]. These authors concluded that the presence of *Hematodinium* caused the host to be susceptible to these infections because they only found the fungus in *Hematodinium*-affected crabs. They also discussed the possibility that host immunosuppression caused by the presence of *Hematodinium* left these animals susceptible to this and other secondary infections. To investigate this putative interaction further, Smith et al. [33] challenged *Hematodinium*-infected and uninfected crabs with the fungus (now identified as a member of the *Ophiocordyceps* clade). Unexpectedly, the presence of *Hematodinium* apparently caused a reduction in the multiplication of the fungus in the haemocoel in comparison to *Hematodinium*-free crabs. However, all crabs, regardless of their infection status, died as a result of the fungal infection. Furthermore, the fungal infection was found in small numbers of edible crabs in the wild that were uninfected by *Hematodinium*, reflecting that the association between the two pathogens was not obligate [33]. These co-infections also reveal that crustaceans infected by *Hematodinium* are able to mount an efficient immune response to other pathogens that is not apparently different to that seen in uninfected hosts. Hence, these observations advocate that *Hematodinium* does not elicit general immune suppression. However, a recent study in which Chinese swimming crabs (*Portunus trituberculatus*) were challenged with *Hematodinium*, has provided evidence of an apparent immunosuppressive action by these parasites [34]. The study revealed that artificially infected crabs showed temporal changes in prophenoloxidase gene expression post-challenge. The prophenoloxidase activating system is a key component of the crustacean immune response (Box 1) [26], and therefore, changes in this are likely to adversely affect host survival. Although the authors concluded that *Hematodinium* showed the ability to cause potential inhibition of the prophenoloxidase activating system, levels of enzyme (i.e., phenoloxidase) activity varied post-challenge from no significant changes through to both elevation and suppression at the different time points studied.

To further investigate the possibility that *Hematodinium* causes generalised immunosuppression, we challenged juvenile edible crabs, with or without low-severity (i.e., early-phase) *Hematodinium* infections, with the non-pathogenic bacterium *Bacillus subtilis*, and its clearance from circulation was monitored by standard plate counts. These experiments demonstrated that crabs with low-severity *Hematodinium* infections showed no changes in their ability to clear the bacteria from circulation in comparison to uninfected crabs (Fig 2B). This implies that crabs with early-phase *Hematodinium* infections are not immunocompromised towards other potential bacterial pathogens. Overall, it is our opinion that generalised immune suppression is unlikely to be the main strategy utilised by *Hematodinium* to overwhelm its host.

The apparent lack of any cell–mediated response directed to the presence of large numbers of *Hematodinium* in the haemolymph points to the possibility that this parasite utilises molecular mimicry or evasion to “hide” from the host’s immune system. While there may be other strategies employed, this would seem the most likely explanation (Table 1). Several host–parasite relationships in which molecular mimicry or specific evasion operates have been investigated using invertebrate models and these provide us with mechanistic explanations that may shed light on the current crustacean–*Hematodinium* interaction. Two good examples of such models include the human malaria parasite, *Plasmodium*, in its invertebrate vectors, the anopheline mosquitoes [35,36], and *Schistosoma mansoni* in the freshwater snail, *Biomphalaria glabrata* [37,38].

Lectins are key immune recognition molecules in a number of invertebrates, which operate by binding unusual carbohydrate residues associated with pathogens and parasites [39]. Larval schistosomes may share glycans with the host haemocytes, leaving these cells unable to recognise the presence of such parasites via lectin-based recognition systems [37,38]. Probing for the presence of shared surface carbohydrates between parasites and haemocytes using biotinylated lectins has proven to be a useful and rapid initial approach to look for their role in molecular mimicry [39]. Such approaches may be a useful first point of investigation in the crustacean–*Hematodinium* model.

## Conclusion

In this Opinion article, we have reviewed and discussed evidence that gives new insight into the host relationship of this key dinoflagellate parasite. This assemblage, belonging to the genus *Hematodinium*, has the capacity to successfully invade and multiply in the tissues of many different crustacean hosts without eliciting any obvious cellular immune response such as parasite encapsulation. There are very few parasites or pathogens that have the ability to circumvent the cellular defence reactions of several different hosts, making this of wide interest to immunologists. Whatever the mechanism(s) employed, these parasites have emerged as a significant threat to the future sustainability of shellfish fisheries and aquaculture worldwide, and hence, they warrant research focus.
